# Anti-Carbamylated Fibrinogen Antibodies Might Be Associated With a Specific Rheumatoid Phenotype and Include a Subset Recognizing *In Vivo* Epitopes of Its γ Chain One of Which Is Not Cross Reactive With Anti-Citrullinated Protein Antibodies

**DOI:** 10.3389/fimmu.2021.733511

**Published:** 2021-10-07

**Authors:** Pauline Brevet, Claire Lattard, Clément Guillou, Pascal Rottenberg, Patrice Fardellone, Xavier Le-Loët, Thierry Lequerré, Pascal Cosette, Olivier Boyer, Manuel Fréret, Olivier Vittecoq

**Affiliations:** ^1^ Rouen University Hospital, Department of Rheumatology & CIC-CRB1404, Rouen, France; ^2^ Normandie University, UNIROUEN, INSERM, U1234, Rouen, France; ^3^ Rouen University Hospital, Department of Pharmacology, Rouen, France; ^4^ Normandie University, UNIROUEN, PISSARO Proteomics Facility & PBS-UMR6270 CNRS, IRIB, Rouen, France; ^5^ Amiens University Hospital, Department of Rheumatology, Amiens, France; ^6^ Rouen University Hospital, Department of Immunology, Rouen, France

**Keywords:** very early rheumatoid arthritis, anti-carbamylated protein antibodies (anti-CarP), fibrinogen, ACPA, prognosis

## Abstract

To identify the targets recognized by anti-carbamylated protein antibodies (anti-CarP) in patients with early Rheumatoid Arthritis (RA), to study the cross-reactivity between anti-CarP and anti-citrullinated protein antibodies (ACPA) and to evaluate their prognostic value. 331 patients (184 RA and 147 other rheumatisms) from the Very Early Arthritis (VErA) French cohort were analyzed. We performed mass spectrometry analysis of RA sera displaying anti-CarP activity and epitope mapping of the carbamylated fibrinogen γ chain to identify immunodominant peptides. The specificity of these targets was studied using competition assays with the major antigens recognized by ACPA. The prognostic value of anti-carbamylated fibrinogen IgG antibodies (ACa-Fib IgG) was compared to that of anti-cyclic citrullinated peptide antibodies (anti-CCP) and anti-CarP using an in-house ELISA. Besides the α chain, the γ chain of fibrinogen, particularly one immunodominant epitope that has a specific reactivity, was identified as a circulating carbamylated target in sera. The prevalence of ACa-Fib was 37% at baseline and 10.9% for anti-CCP-negative RA. In anti-CCP-negative patients, ACa-Fib positivity was associated with a more inflammatory and erosive disease at baseline but not with rapid radiological progression, which remains strongly related to anti-CCP antibodies. Fibrinogen seems to be one of the antigens recognized *in vivo* by the anti-CarP response, particularly 2 epitopes of the γ chain, one of which is not cross reactive with ACPA. This specificity might be associated with a distinct clinical phenotype since ACa-Fib IgG were shown to be linked to systemic inflammation in very early RA but not to rapid radiological progression.

## Introduction

Anti-citrullinated protein antibodies (ACPA), the hallmark of rheumatoid arthritis (RA), are of both diagnostic and prognostic interest (1, 2). Besides citrulline, other post-translational modifications (PTMs) are currently being studied, including carbamylation. In 2011, Shi et al. detected anti-carbamylated protein antibodies (anti-CarP) in the serum of RA patients, 45% of IgG isotype and 43% of IgA isotype. These antibodies might display diagnostic value since, among ACPA-negative patients, 16% and 30% had anti-CarP antibodies of IgG and IgA isotype respectively (3). They also have a prognostic value since their presence signals a form of RA with more severe radiological damage (4). Carbamylated Fetal Calf Serum (FCS) is the substrate currently used for enzyme-linked immunosorbent assay (ELISA) tests, which represents the gold standard. However, FCS contains many antigenic targets that remain undetermined. To better understand the clinical interest of anti-CarP, it seems relevant to identify proteins that are spontaneously carbamylated *in vivo.* Among the antigens tested (vimentin, GRP78, albumin, etc.) (5–7), carbamylated fibrinogen was the most recognized by these antibodies (8, 9). Moreover, Jones et al. showed that the anti-carbamylated fibrinogen response primarily targeted the fibrinogen β-chain (10). Finally, some studies suggest that there is cross-reactivity between ACPA and anti-CarP (11, 12).

The primary objective of this study was to identify the targets recognized by anti-CarP in the in the serum of RA patients. Secondary objectives were to determine whether the anti-carbamylated fibrinogen (ACa-Fib) antibody response was specific and different to anti-cyclic citrullinated peptide (anti-CCP) antibody response, in other words, to study the cross-reactivity between ACa-Fib and anti-citrullinated fibrinogen antibodies, and to evaluate their prognostic value in the Very Early Arthritis (VErA) cohort.

## Patients and Methods

### Patients and Serum Samples

VErA is a multicenter cohort that prospectively included patients with early inflammatory rheumatism. The inclusion criteria were that participants were at least 18 years old and had at least 2 swollen joints for at least 4 weeks, which had been evolving for less than 6 months (median symptom duration of 4 months). Patients had to be corticosteroid and disease-modifying anti-rheumatic drug (DMARD) naïve. Patients with inflammatory back pain were excluded. A biological assessment and X-rays of the wrists, hands, and forefeet (and all other painful joints) were performed at inclusion and regular intervals. Patients were followed up every 6 months for 10 years. A total of 331 patients were included from 1998 to 2002, 184 patients with RA and 147 patients classified as having “other rheumatic diseases” (systemic lupus erythematosus, ANCA associated vasculitis, Gougerot Sjögren’s syndrome, systemic sclerosis, psoriatic arthritis, mixed connective tissue diseases, crystal-induced arthritis) or “undifferentiated rheumatic disease”. Sera used as controls (n=100) were obtained from a serum bank of healthy individuals at the Établissement Français du Sang (EFS). For the clinical part of the present study, only sera from the 184 RA patients meeting the ACR 2010 criteria (which were applied when available) were used (13). This study was approved by the Upper Normandy Ethics Committee (file: 95/138/HP).

### Proteomic Analysis: Enzymatic Digestion of Proteins and Nano LC-MS/MS Analysis

To perform proteomic analysis from VErA sera samples known to be ACa-FCS-positive, protein concentration was determined using the standard Bradford method (Biorad). Enzymatic digestion of proteins in sera samples has been described previously (14). Identification of carbamylated peptides was performed with a classic setup of mass spectrometry analysis using a Q-exactive Plus (ThermoScientific) equipped with a nanoESI source, as described previously (15). All spectra obtained were exported in “raw” format to identify peptides and proteins with Proteome Discoverer 1.4 software (Thermo Scientific). Peak lists were searched using MASCOT search software (Matrix Science) against the *human* Swissprot database with the following parameters: one miss cleavage site allowed, carbamidomethylation on cysteine, oxidation on methionine, and carbamylation on lysine as variable modifications. The parent-ion and daughter-ion tolerances were 5ppm and 0.02Da respectively.

Proteomic data deposit on ProteomeXchange *via* the PRIDE database (Project accession: PXD028121).

### Antigens and Carbamylation

Fetal calf serum (Sigma-Aldrich) and peptides of the γ chain of fibrinogen were incubated with potassium cyanate 1 M (Sigma-Aldrich) at 4 mg/mL and 2 to 4 mg/mL respectively during 12 hours at 37°C and dialyzed against water. Human fibrinogen, purified from human plasma (F3879, Sigma-Aldrich), was incubated with potassium cyanate 0.5 M for 3 days and dialyzed against water. Carbamylation of protein/peptide was determined by ELISA (OxiSelec Protein Carbamylation Sandwich ELISA Kit, Cell Biolabs).

### Detection of Anti-CarP Antibodies of IgG Isotype

Detection of IgG autoantibodies recognizing carbamylated FCS (ACa-FCS), carbamylated fibrinogen (ACa-Fib), and carbamylated peptides of the γ chain of fibrinogen (ACa-Fib γ chain) was performed using home-made ELISA. Carbamylated and native proteins/peptides were coated at 10 µg/mL in carbonate-bicarbonate buffer on Nunc Maxisorp plates (Thermo Scientific) and incubated overnight at 4°C. For blocking, plates were incubated with PBS BSA 1% for 5 hours at 4°C. Sera from healthy controls (from Etablissement Français du Sang, EFS) or patients were diluted (1/50) in PBS BSA 1% Tween 0.05% and incubated overnight. Plates were incubated with biotinylated anti-human IgG (SouthernBiotech) diluted (1/3000) in PBS BSA 1% Tween 0.05% for 1h15 and HRP-conjugated streptavidin (1/25000) (Thermo Scientific) for 30 minutes at 4°C before the addition of tetramethylbenzidine substrate solution and STOP buffer (Sigma-Aldrich). Washing Washing with PBS Tween 0.05% was carried out between steps. The reactivity to native proteins/peptides was subtracted from that of the corresponding carbamylated protein/peptide. The absorbance was measured at 415 nm and converted to arbitrary units (AU): we performed a range with an anti-carbamylation (homocitrulline) monoclonal antibody (Cayman Chemical) (serial dilution at ½ with a first range point dilution at 1/2000) with the same protocol as previously described. The threshold of positivity was determined at 38.12 AU for ACa-FCS IgG (healthy controls (n=100) mean + 3 standard deviations) and 19.47 AU for ACa-Fib IgG (after performing a ROC curve using data obtained from healthy controls (n=100) and RA patients (n=184) of the VErA cohort).

### Detection of Anti-CCP Antibodies

Detection of anti-CCP antibodies was performed using second generation commercially available kits (EuroImmun). Anti-CCP antibodies levels ≥ 10 arbitrary units (AU) were considered as positive.

### Epitope Mapping of the γ Chain of Carbamylated Fibrinogen

Fifteen peptides of the γ chain of fibrinogen were synthesized by Eurogentec (27 amino acids overlapping on 3 amino acids, containing at least one lysine residue) and reconstituted according to the protocol described above (**Table 1**). ACa-Fib γ chain peptides were detected according to the previously described protocol. Sixteen positive sera of RA patients with ACa-Fib and 30 sera of healthy controls (EFS) were tested. The threshold of positivity was determined at 0.25 OD (Optical Density), which was the cut off used in other studies (3).

**Table 1:** Native or carbamylated peptides of fibrinogen γ chain (27 mer-straddling 3 amino acids) containing at least one lysine.

Peptide	Sequence	Concentration native form (mg/mL)	Concentration carbamylated form (mg/mL)
1	CGIADFLSTYQTKVDKDLQSLEDILHQ	4	4
2	LHQVENKTSEVKQLIKAIQLTYNPDES	4	4
3	DESSKPNMIDAATLKSRKMLEEIMKYE	4	2
4	KYEASILTHDSSIRYLQEIYNSNNQKI	4	4
5	QKIVNLKEKVAQLEAQCQEPCKDTVQI	4	4
6	VQIHDITGKDCQDIANKGAKQSGLYFI	4	4
7	YFIKPLKANQQFLVYCEIDGSGNGWTV	4	2
8	WTVFQKRLDGSVDFKKNWIQYKEGFGH	4	4
9	FGHLSPTGTTEFWLGNEKIHLISTQSA	2	2
10	AMFKVGPEADKYRLTYAYFAGGDAGDA	4	4
11	GDAFDGFDFGDDPSDKFFTSHNGMQFS	4	2
12	QFSTWDNDNDKFEGNCAEQDGSGWWMN	4	4
13	WMNKCHAGHLNGVYYQGGTYSKASTPN	4	4
14	TPNGYDNGIIWATWKTRWYSMKKTTMK	4	4
15	TMKIIPFNRLTIGEGQQHHLGGAKQVR	4	4

Name, sequences and concentrations.

### Cross-Reactivity Assay With Immunodominant Peptides of α and β Chains of Citrullinated Fibrinogen

Patient sera positive for ACa-Fib γ chain peptides 5 (n=4) and 13 (n=3) were selected for the competition experiments. Carbamylated and native peptides 5 or 13 were coated as described above. Sera were diluted (1/50) in PBS BSA 1% Tween 0.05% and incubated for 2 hours with increasing concentrations (0, 0.1, 0.5, 1, 5 and 10 µg) of peptide 5, peptide 13, immunodominant peptide of citrullinated fibrinogen α chain (GV(cit)GP(cit)VVE(cit)HQSACKDSDWP) or β chain (PSL(cit)PAPPPISGGGY(cit)A(cit)PAK). These two peptides were synthesized by Eurogentec. Arginine residues were replaced by citrulline residues during the synthesis. The protocol for antibody detection is the same as described above.

### Statistics

Prism7 (GraphPad) was used for statistical analysis. The statistical differences in antibody levels between healthy controls and patients were determined by one-way ANOVA and Tukey post-test.

## Results

### Titer, Prevalence, and Distribution of ACa-FCS and Anti-CCP Antibodies in Healthy Donors and the VErA Cohort

**Figure 1:**
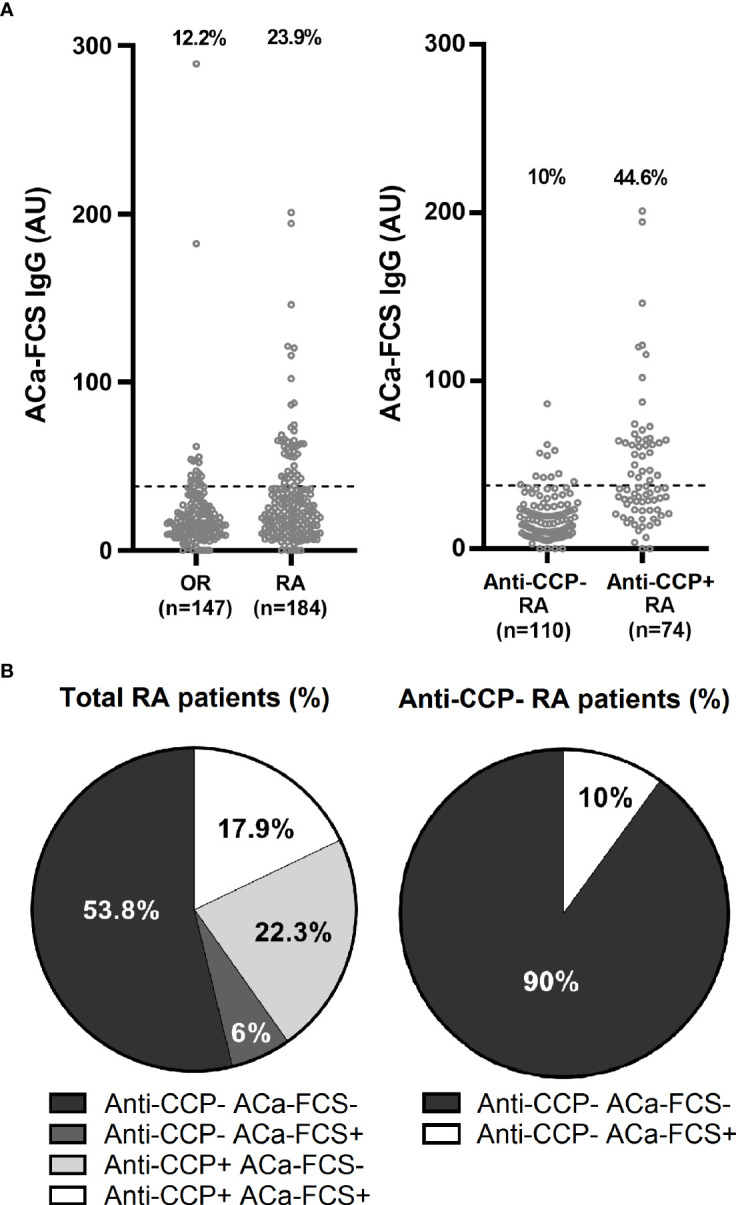
Titer, prevalence, and distribution of ACa-FCS and anti-CCP antibodies in the VErA cohort at baseline. **(A)** ELISA test for detection of ACa-Fib IgG based on RA status (n=184) and other rheumatism (OR) (n=147) and anti-CCP antibodies status in RA patients (n=184). **(B)** Distribution of anti-CCP antibodies and ACa-FCS in RA patients from the VErA cohort. Anti-CCP, anti-cyclic citrullinated peptide antibodies; ACa-FCS, anti-carbamylated fetal calf serum antibodies.

In the healthy population (n=100), the prevalence of ACa-FCS IgG antibodies was estimated at 1% while in RA and other rheumatism (OR) patients of the VErA cohort, it was calculated at 23.9% and 12.2% respectively. The distribution of ACa-FCS positivity according to anti-CCP status is summarized in **Figures 1A, B**.

### One of the Antigens Targeted *In Vivo* by the Anti-CarP Response in RA Is Located in the γ Chain of Fibrinogen

**Figure 2:**
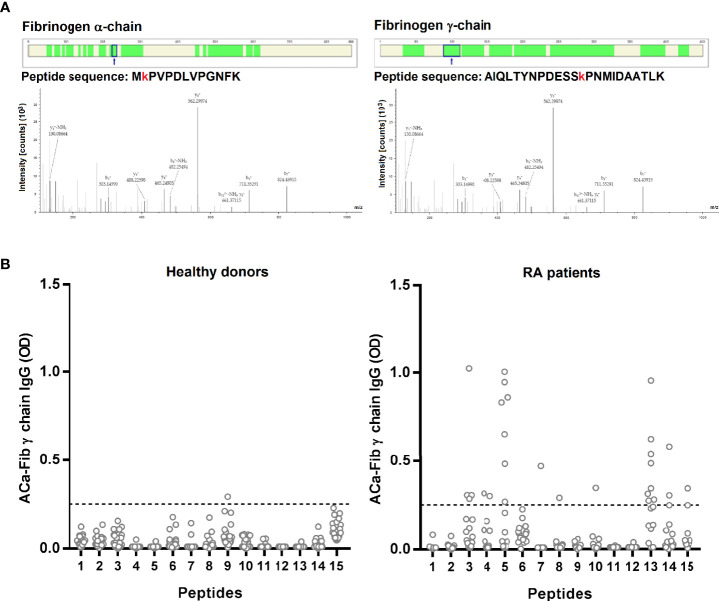
Identification by MS/MS of carbamylated target of anti-CarP *in situ* and epitope mapping of the antibody response directed against the γ chain of carbamylated fibrinogen. **(A)** Identified peptides on the α chain (40% of coverage rate) and γ chain (65% of coverage rate) have a carbamyl residue on lysine. Filters used for identification are as follows: high peptide confidence; peptide rank 1 and a peptide score > 20. **(B)** ELISA test for detection of ACa-Fib γ chain IgG from RA patients with Aca-Fib IgG (n=16) and healthy donors (n=30), expressed as OD (Optical Density) at 450 nm. ACa-Fib γ chain, anti-carbamylated fibrinogen γ chain antibodies. The horizontal line corresponds to the threshold of positivity (OD ≥ 0.25) and each point represents a patient.

To attempt to identify the specific targets *in vivo* of the anti-CarP response, 15 sera of RA patients were selected according to the presence of ACa-FCS antibodies and the detection of carbamylated proteins in the sera. Then, they were analyzed using mass spectrometry. According to this approach, 2 chains of fibrinogen were identified, more precisely the α chain and the γ chain in 13 and 2 sera respectively of patients with very early RA (**Figure 2A**). For these 2 fibrinogen chains, the same peptide sequences, containing a carbamylated lysine residue, were found in these patients.

### Epitope Mapping of Antibody Response Directed Against the γ Chain of Carbamylated Fibrinogen

We performed epitope mapping of the γ chain of carbamylated fibrinogen. We selected RA sera that were immunopositive for ACa-Fib IgG (n=54). Sera were tested by ELISA for reactivity towards the 15 linear peptides of the γ chain of fibrinogen, that were carbamylated *in vitro*. Among these 54 sera, 16 recognized at least one of the 15 peptides of the γ chain of carbamylated fibrinogen (data not shown). These 16 sera reacted with a restricted set of epitopes (**Figure 2B**). Interestingly, it seems that ACa-Fib IgG recognized mostly 2 peptides (peptides 5 and 13). Of the 16 RA patients, 7 and 8 specifically recognized peptides 5 and 13 respectively; 2 sera bound to the 2 peptides while 13 were directed against at least one of the two.

### At Least a Part of the ACa-Fib response Does Not Overlap With the Anti-Citrullinated Fibrinogen Response

**Figure 3:**
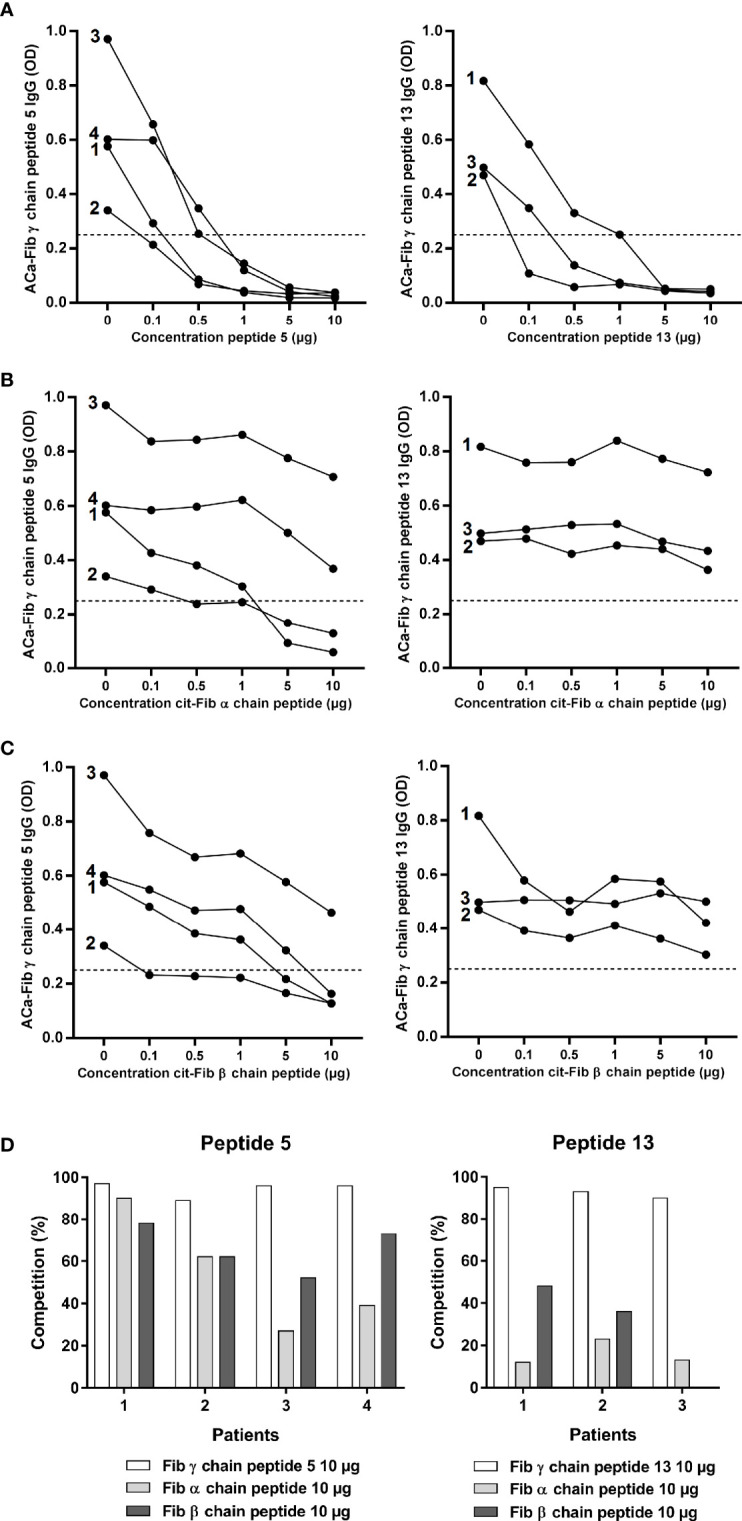
Cross reactivity assay of carbamylated γ chain of fibrinogen peptides 5 and 13 with major epitopes of the anti-citrullinated fibrinogen response. **(A)** ELISA inhibition test of carbamylated γ chain of fibrinogen peptides 5 or 13 with their corresponding peptides at increasing concentrations (0, 0.1, 0.5, 1, 5 or 10 µg/mL). **(B)** ELISA inhibition test of carbamylated γ chain of fibrinogen peptides 5 or 13 with an immunodominant epitope of citrullinated fibrinogen α chain at increasing concentrations (0, 0.1, 0.5, 1, 5 or 10 µg/mL). **(C)** ELISA inhibition test of carbamylated γ chain of fibrinogen peptides 5 or 13 with an immunodominant epitope of citrullinated fibrinogen β chain at increasing concentrations (0, 0.1, 0.5, 1, 5 or 10 µg/mL). **(D)** Percentages of inhibition for each peptide. ELISA tests expressed as OD (Optical Density) at 450 nm. Sera highly positive for carbamylated γ chain of fibrinogen peptide 5: n=4; sera highly positive for carbamylated γ chain of fibrinogen peptide 13: n=3. ACa-Fib γ chain, anti-carbamylated fibrinogen γ chain antibodies.

ACa-Fib response, in part, targeted peptides 5 and 13 of the γ chain of carbamylated fibrinogen. Based on inhibition tests with their corresponding peptides, we investigated whether there was cross-reactivity with the most representative immunodominant peptides of the anti-citrullinated response, as suggested in some studies (11). We analyzed the ability of peptides 5 and 13 of the γ chain of carbamylated fibrinogen to compete at increasing concentrations up to saturating concentrations of 10 µg/ml binding to themselves (positive control) using the sera of RA patients (n=4 and n=3 for peptide 5 and 13 respectively) who had a strong reactivity towards either of these 2 peptides (**Figure 3A**). Each peptide completely inhibited its binding to RA sera from a saturating concentration of 5µg/ml. The same competition experiment was performed with the immunodominant peptide of the citrullinated fibrinogen α chain (cit-Fib α chain peptide) (**Figure 3B**) and the citrullinated fibrinogen β chain (cit-Fib β chain peptide) (**Figure 3C**). There was cross-reactivity between peptide 5 of the γ chain of carbamylated fibrinogen and an α chain peptide of citrullinated fibrinogen. A saturating dose of 10 µg/ml of cit-Fib α chain peptide resulted in a strong inhibition of >30% of the 4 sera tested (**Figure 3D**). In contrast, a weak inhibition was observed for the cit-Fib α chain peptide on peptide 13 (**Figure 3B**). This weak inhibition represents a percentage of <25% for one of the 3 sera tested and <15% for the two other sera (**Figure 3D**). There was also cross-reactivity between γ chain peptide 5 of carbamylated fibrinogen and cit-Fib β chain peptide (**Figure 3C**), the binding of three out of four sera was inhibited at the saturating dose of 10 µg/ml. This degree of competition is important because all sera had at least a percentage inhibition of > 50% (**Figure 3D**). In contrast, concerning peptide 13, inhibition by the cit-Fib β chain peptide seems less important than for peptide 5 because the binding of the 3 sera tested was not completely inhibited at a concentration of 10 µg/ml (**Figure 3C**). Furthermore, for the serum of two patients, this inhibition was <50% and the other serum was not inhibited at all by the cit-Fib β chain peptide (**Figure 3D**). These data suggest that peptide 13 (which we will name Ca-Fibγ361-387) contains a specific epitope of the anti-carbamylated fibrinogen response that is able to lead to an immunization distinct from citrullination.

### Titer, Prevalence, and Distribution of ACa-Fib Antibodies in Healthy Donors and the VErA Cohort According to Anti-CCP and/or ACa-FCS Status

**Figure 4:**
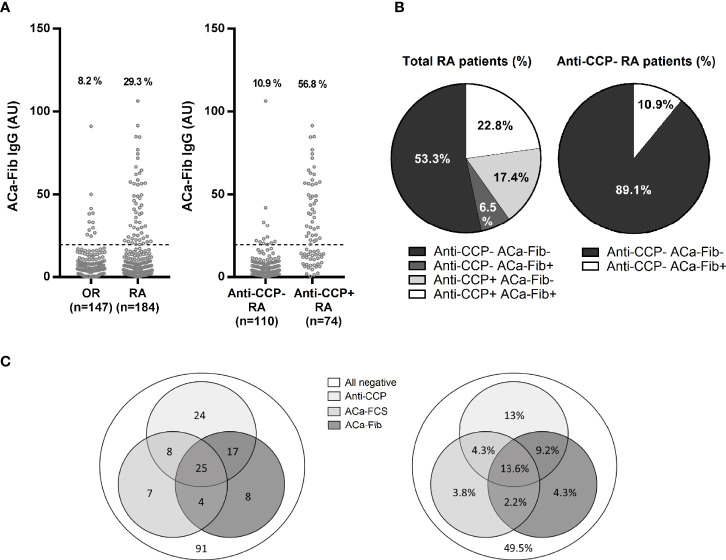
Titer, prevalence and distribution of ACa-Fib and anti-CCP antibodies in the VErA cohort at baseline and distribution according to the triple status ACa-Fib, ACa-FCS, and anti-CCP antibodies. **(A)** ELISA test for detection of ACa-Fib IgG based on RA status (n=184) and other rheumatism (n=147) and anti-CCP antibodies status in RA patients (n=184). **(B)** Distribution of anti-CCP antibodies and ACa-FCS in RA patients from the VErA cohort. **(C)** Distribution of immunological profile in RA patients considering triple status anti-CCP antibodies, ACa-Fib, ACa-FCS. Values are expressed in absolute numbers and percentages and represent the positivity of each auto-antibody. Anti-CCP, anti-cyclic citrullinated peptide antibodies; ACa-Fib, anti-carbamylated fibrinogen antibodies; ACa-FCS, anti-carbamylated fetal calf serum antibodies.

The α and γ chains of carbamylated fibrinogen might be two targets of the anti-CarP response in RA. Jones et al. showed that this response was also directed against the fibrinogen β chain. Thus, the 3 chains making up fibrinogen appear to comprise immunodominant epitopes of the anti-CarP response in RA. Thus, we developed an ELISA using the whole fibrinogen protein as a substrate to detect ACa-Fib IgG. While no ACa-Fib IgG antibodies were measured in the healthy population, our ELISA test revealed that patients classified as having RA and OR in the VErA cohort were ACa-Fib IgG-positive in a proportion of 29.3% and 8.2% respectively. Considering all RA in the cohort, 56.8% and 10.9% of patients were immunopositive for ACa-Fib IgG in anti-CCP-positive and anti-CCP-negative patients respectively (**Figure 4A**). The serological profiles for both populations of autoantibodies in the VErA cohort are illustrated in **Figure 4B**. Of all patients with very early RA, 53.3% were immunonegative for both anti-CCP antibodies and ACa-Fib, 6.5% were immunopositive for ACa-Fib, 17.4% were immunopositive for anti-CCP antibodies, and 22.8% were positive for both anti-CCP antibodies and ACa-Fib. Among anti-CCP negative patients, 10.9% of sera were ACa-Fib immunopositive. Although these profiles were close to those obtained with the ELISA test using carbamylated FCS as a substrate (**Figure 1B**), these findings were not strictly similar. The autoantibody profiles with ACa-FCS, ACa-Fib, and anti-CCP status in RA patients from the VErA cohort are shown in **Figure 4C**. Values are expressed in absolute numbers and percentages and represent the positivity of each autoantibody. Of the RA patients who were seronegative for anti-CCP antibodies at baseline (n=115), 11 and 12 patients had ACa-FCS and ACa-Fib IgG respectively. When carbamylated FCS was used as a substrate for the ELISA test, some epitopes of carbamylated fibrinogen were not recognized (n=8 patients in the cohort). Conversely, when carbamylated fibrinogen was used as a substrate for the ELISA test, other carbamylated targets within the FCS were not detected in 7 patients of the cohort.

### Clinical Interest of ACa-Fib Autoantibodies and Their Role in Prognostic Strategies in Relation to Anti-CCP Antibodies and ACa-FCS

**Figure 5:**
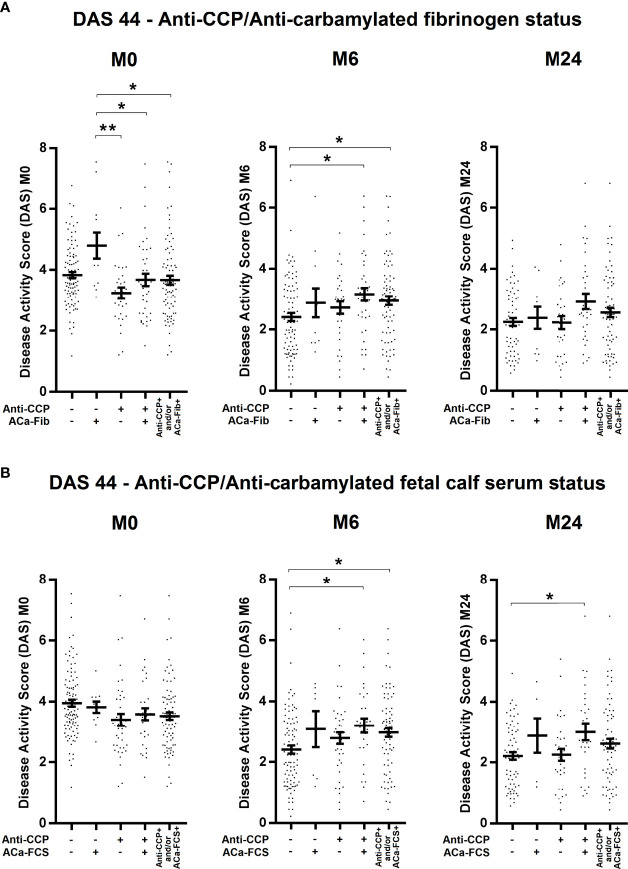
Evolution of disease activity of RA patients at baseline, 6 months, and 2 years according to anti-CCP antibodies and ACa-Fib status or ACa-FCS status. Disease Activity Score (DAS) 44 of RA patients according to **(A)** Anti-CCP/ACa-Fib status or **(B)** Anti-CCP/ACa-FCS status at baseline (M0), 6 months (M6), and 2 years (M24). Averages with SEM are shown. Groups compared using one-way ANOVA and Tukey post-test, *p-value<0.05, **p-value<0.01. Anti-CCP, anti-cyclic citrullinated peptide antibodies; ACa-Fib, anti-carbamylated fibrinogen antibodies; ACa-FCS, anti-carbamylated fetal calf serum antibodies.

**Figure 6:**
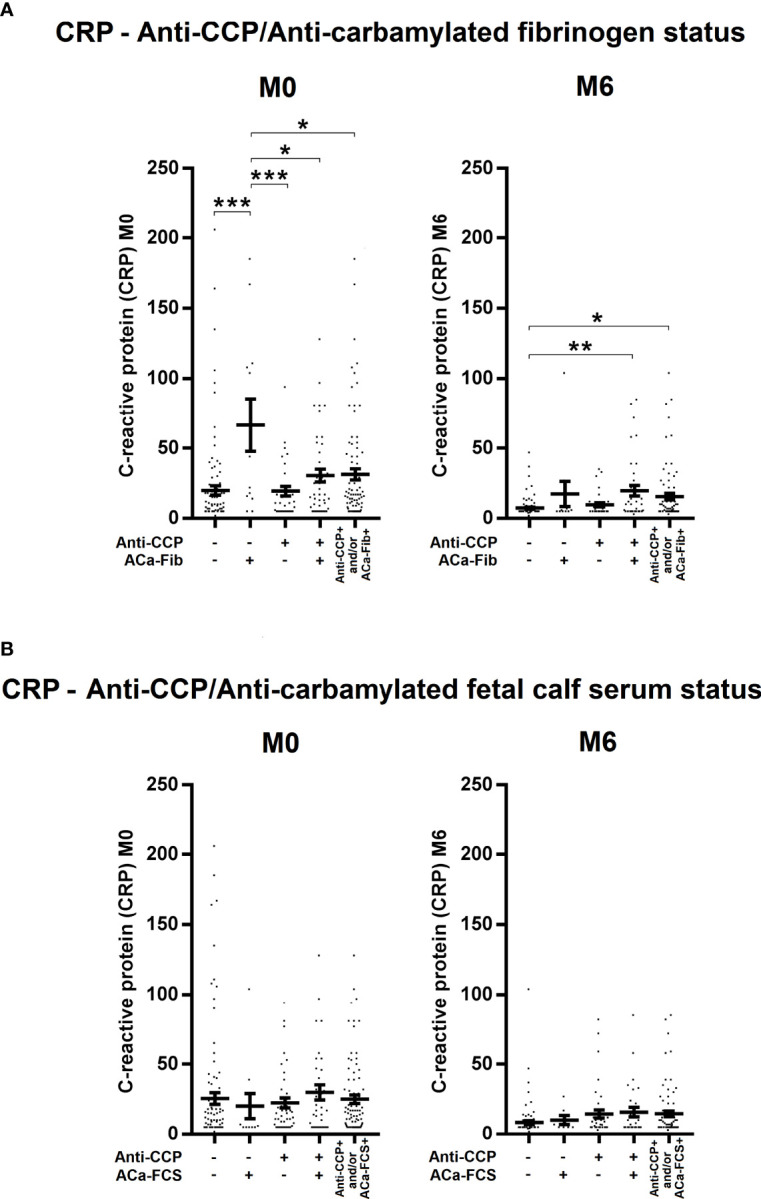
Evolution of systemic inflammation of RA patients at baseline and 6 months according to anti-CCP antibodies and ACa-Fib or ACa-FCS status. C reactive protein (CRP) levels of RA patients according to **(A)** Anti-CCP/ACa-Fib status or **(B)** Anti-CCP/ACa-FCS status at baseline (M0), 6 months (M6), and 2 years (M24). Averages with SEM are shown. Groups were compared using one-way ANOVA and Tukey post-test, *p-value<0.05, **p-value<0.01, ***p-value<0.001. Anti-CCP, anti-cyclic citrullinated peptide antibodies; ACa-Fib, anti-carbamylated fibrinogen antibodies; ACa-FCS, anti-carbamylated fetal calf serum antibodies.

To evaluate potential clinical interest in ACa-Fib IgG from 184 RA patients of the VErA cohort who met the 2010 ACR/EULAR criteria, three follow-up time-points were considered, i.e., baseline (M0), 6 months (M6), and 2 years (M24). The DAS (Disease Activity Score) 44 (index based on 44 joints and including levels of ESR) was calculated and its mean values are shown in **Figure 5**. In the early stages of RA, a small subgroup of patients with only ACa-Fib (n=12) was characterized by a significantly higher disease activity than that observed with other autoimmune profiles (**Figure 5A**). However, this link between ACa-Fib and DAS 44 was only observed prior to DMARD initiation. The DAS 44 is a composite index reflecting both joint activity and systemic inflammation. In this respect, we wondered whether the relationship between disease activity and ACa-Fib IgG positivity at baseline was due to systemic inflammation. This latter was determined by C reactive protein (CRP) levels (**Figure 6**). Once again, compared to ACa-FCS status, CRP values at the onset of RA were higher in the ACa-Fib-positive subgroup than those measured in the other subsets, notably characterized by single positivity of anti-CCP antibodies (p<0.001) (**Figure 6A**). Finally, no link between ACa-FCS and DAS44 was found at any time points (**Figure 5B**) and this also applied to CRP levels (**Figure 6B**). Compared to double negative patients, those who were immunopositive for anti-CCP antibodies and/or ACa-FCS antibodies had a significantly higher DAS 44 (**Figure 5B**). However, such a relationship was not found with CRP levels, which means that seropositivity of anti-CCP antibodies and/or ACa- FCS autoantibodies are related to disease articular activity rather than systemic inflammation. These data are consistent with those of Truchetet et al. (4).

**Figure 7:**
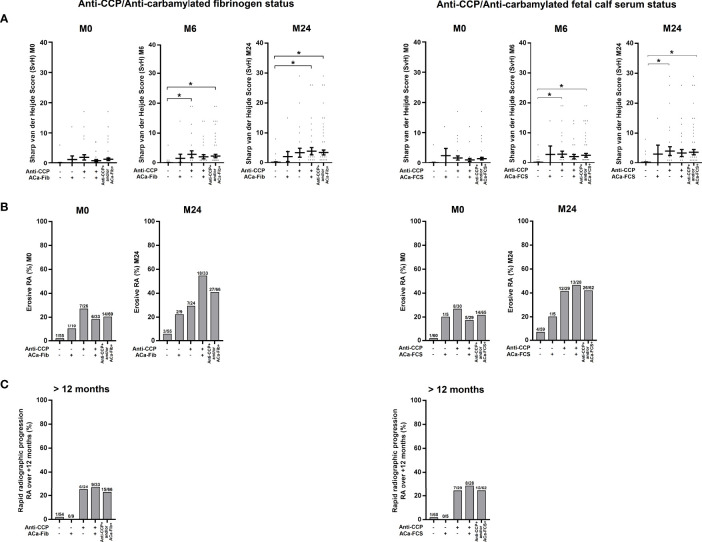
Evolution of structural parameters of RA patients according to anti-CCP antibodies and ACa-Fib or ACa-FCS status. **(A)** van der Heijde modified Sharp score at baseline (M0), 6 months (M6), and 2 years (M24) according to Anti-CCP/ACa-Fib status or Anti-CCP/ACa-FCS status. Averages with SEM are shown. Groups were compared using one-way ANOVA and Tukey post-test, *p-value<0.05. **(B)** Percentage of erosive RA at baseline (M0) and 2 years (M24) according to Anti-CCP/ACa-Fib status or Anti-CCP/ACa-FCS status. **(C)** Percentage of patients with rapid radiographic progression over a period of 12 months starting from M0 according to Anti-CCP/ACa-Fib status or Anti-CCP/ACa-FCS status. Anti-CCP, anti-cyclic citrullinated peptide antibodies; ACa-Fib, anti-carbamylated fibrinogen antibodies; ACa-FCS, anti-carbamylated fetal calf serum antibodies.

Structural impairment of RA patients was assessed according to data on *Van der Heijde* modified Sharp scores that were collected at M0, M6, and M24, the erosive status at M0 and M24 and the degree of structural progression and more precisely rapid radiological progression defined by a 5-point change in the total Sharp score over the first year of evolution. As shown in **Figure 7**, all ACPA-positive subgroups, regardless of ACa-FCS and/or ACa-Fib status, had a higher proportion of erosive RA at M24 and were associated with rapid radiological progression. The small ACa-Fib-positive subgroup which had more inflammatory disease at RA onset showed a tendency to be not associated with rapid radiological progression.

## Discussion

The characteristics of the ACa-FCS profile obtained in our regional VErA cohort are comparable to those found in the French national cohort ESPOIR (*Etude et Suivi des POlyarthrites Indifférenciées Récentes*), which comprised 720 patients with the same inclusion criteria (4). Indeed, 36% and 32% of patients in these 2 cohorts, regardless of their RA or OR diagnosis, were immunopositive for ACa-FCS respectively, supporting the external validity of our results (**Figure 1**). When we consider ACa-Fib, their prevalence in VErA is 37%, whatever the patient’s status (RA or not), which is similar to ACa-FCS (targeting multiple, not clearly identified, antigens). This has reinforced the idea that fibrinogen is probably one of the main targets of the anti-CarP response in RA. When anti-CarP fine specificity is studied, Brink et al. have shown there was a clear increase of antibodies against fibrinogen (Fibß36-52) during the early stage of the disease compared to other PTMs (9).

We were able to directly demonstrate with mass spectrometry the presence of an *in vivo* carbamylated target by analysis of RA sera, known to be ACa-FCS-positive and to contain carbamylated proteins (**Figure 2A**). In addition to the α and β chains of fibrinogen that are already known as targets of citrullination and carbamylation in RA, we have identified a new target, the γ chain of fibrinogen, that contains 2 immunodominant peptides, peptide 5 and peptide 13 (Ca-Fibγ361-387) (**Figure 2B**). However, the fact that fibrinogen was detected in a carbamylated format in the circulation does not mean that the protein is complexed to autoantibodies since the anti-CarP response was shown to be of overall low avidity as compared to that of ACPA-IgG (16). Furthermore, our findings suggest that the pool of carbamylated antigens present in FCS does not include all epitopes of carbamylated fibrinogen and that other carbamylated proteins are targeted in this biological fluid. In this regard, another team has identified, using immunochemistry or mass spectrometry analysis, the presence of a large number of carbamylated proteins in synovial tissue and synovial fluid, that could be recognized by anti-CarP antibodies present in the sera of RA patients (17, 18).

The fact that the response to the γ chain is restricted to two main epitopes suggests that the amino acids surrounding homocitrulline play a key role in this recognition, as shown for ACPA that targets citrulline residues located in a particular amino acid environment (19). Nevertheless, among patients with ACa-Fib, a high proportion (38/54) did not recognize any of these peptides, suggesting that some epitopes may be conformational and/or that the other targets are located on the α and β chains of fibrinogen, as suggested previously (10).

We focused our study on the γ chain of fibrinogen which is a different target of citrullination (20). There are many data suggesting cross-reactivity between ACPAs and anti-CarP (21). In this regard, our competition experiments performed with the immunodominant peptides of the anti-citrullinated fibrinogen response have shown that only ACa-Fib that recognize peptide 13 of the γ chain (Ca-Fibγ361-387) has a specific reactivity distinct from anti-citrullinated fibrinogen (**Figure 3**). This finding could be explained by the fact that its sequence does not contain any arginine residue that could be the site of citrullination. This point is of particular relevance to better understand the profile of RA serum autoantibodies against the citrullinome and the homocitrullinome. In this regard, despite a lower reactivity of RA serum samples against homocitrullinated peptides using a high density peptide array to screen the entire proteome, Lo et al. showed that extensive anti-CarP antibody reactivity was seen in a restricted set (4/18) of RA samples (22). Although these RA sera also showed ACPA reactivity and did not constitute an anti-CarP-positive and ACPA-negative subgroup, it is important to remember that this study defined ACPA as a family of autoantibodies likely to recognize a larger panel of citrullinated peptides in which some of them are not targeted by anti-CCP antibodies, whose main antigen is citrullinated fibrinogen, including a large number of proteins that are not known to be associated with RA.

Even if we have discovered a new target, distinct from ACPA response, it is important to recall that multiple antigenic targets are recognized by ACPA, anti-CarP, and more recently by anti-acetylated protein antibodies with broad reactivity to various antigens (21, 23). Indeed, several investigations suggest that monoclonal ACPA antibodies can exhibit multireactivity that extends to other PTMs (carbamylation, acetylation) with a hierarchy of these reactivities comprising multireactivity restricted to citrulline, multireactivity to citrullinated and carbamylated antigens, and multireactivity that covers all 3 PTMs. For some of these clones, it has been demonstrated that the carbamylated protein or acetylation protein binding is of higher apparent affinity than citrullinated protein binding. Taken together, all these findings suggest that anti-CarP should be considered as a different entity to ACPA/anti-modified proteins antibodies profiles (24).

In the VErA cohort, we have identified a small subgroup of RA patients, who are anti-CCP-negative/ACa-Fib-positive and who have a higher disease activity at disease onset than patients who are immunopositive for anti-CCP antibodies (**Figure 5A**). Our data suggest that this relationship is rather due to systemic inflammation than to joint activity. Thus, ACa-Fib antibodies are possibly associated with initial systemic inflammation (**Figure 6A**). This link between ACa-Fib and CRP levels prior to DMARD initiation raises the question of a potential relationship with structural damage since systemic inflammation is often associated with a higher risk of radiological progression (25). Previous studies showed that the presence of ACa-FCS was associated with more severe radiological damage in RA, regardless of anti-CCP status but also in the subgroup of anti-CCP-negative/ACa-FCS-positive patients who displayed a significantly higher *van der Heijde* modified total Sharp score compared to anti-CCP-negative and ACa-FCS-negative patients (**Figure 7**). In addition, Ajeganova et al. tested the added value of ACa-FCS to ACPA and RF serological status in matrices predicting joint destruction. This did not make it possible to better rank the severity of radiological damage at 5 years compared to the information provided by ACPA alone (26). In our study, when patients were stratified according to autoantibody profile in relation to anti-CCP antibodies, ACa-FCS and ACa-Fib, rapid radiological progression was associated with the positivity of anti-CCP antibodies and not with the presence of anti-CarP (**Figure 7**).

To conclusion, in the specific subgroup of patients negative for ACPA for whom we lack biomarkers to predict the structural outcome, the presence of anti-CarP, more specifically in ACa-Fib-positive patients, might be related to a more benign form since it is not related to rapid radiological progression but is characterized by a more active disease at presentation. Due to the limited size of the different subgroups that were defined according to anti-CCP/anti-CarP profiles, this potential relationship between anti-ACa-Fib antibodies and initial systemic inflammation needs to be replicated in a larger cohort of very early arthritis prior to drawing robust conclusions.

## Data Availability Statement

The datasets presented in this study can be found in online repositories. The names of the repository/repositories and accession number(s) can be found below: PRIDE, PXD028121.

## Ethics Statement

The studies involving human participants were reviewed and approved by Upper Normandy Ethics Committee (file: 95/138/HP). The patients/participants provided their written informed consent to participate in this study.

## Author Contributions

OV and MF designed and supervised the project. XL-L, PF, and OV supervised the VErA cohort. PB, CL, CG, PR, and MF performed the experiments. PB, CL, CG, PR, TL, OB, MF, PC, and OV analyzed the data. PB, MF, and OV wrote the manuscript with the help of all authors. All authors contributed to the article and approved the submitted version.

## Funding

This work was supported by a grant from MSD and Novartis (DREAMER bursary) and financial support from Fondation Arthritis. The funders were not involved in the study design, collection, analysis, interpretation of data, the writing of this article or the decision to submit it for publication.

## Conflict of Interest

The authors declare that the research was conducted in the absence of any commercial or financial relationships that could be construed as a potential conflict of interest.

## Publisher’s Note

All claims expressed in this article are solely those of the authors and do not necessarily represent those of their affiliated organizations, or those of the publisher, the editors and the reviewers. Any product that may be evaluated in this article, or claim that may be made by its manufacturer, is not guaranteed or endorsed by the publisher.
